# Healthfulness of Foods Advertised in Small and Nontraditional Urban Stores in Minneapolis–St. Paul, Minnesota, 2014

**DOI:** 10.5888/pcd13.160149

**Published:** 2016-11-10

**Authors:** Timothy L. Barnes, Jennifer E. Pelletier, Darin J. Erickson, Caitlin E. Caspi, Lisa J. Harnack, Melissa N. Laska

**Affiliations:** Author Affiliations: Jennifer E. Pelletier, Darin J. Erickson, Lisa J. Harnack, Melissa N. Laska, University of Minnesota, Division of Epidemiology and Community Health, Minneapolis, Minnesota; Caitlin E. Caspi, University of Minnesota, Department of Family Medicine and Community Health, Minneapolis, Minnesota.

## Abstract

**Introduction:**

Shopping at small food stores, such as corner stores and convenience stores, is linked with unhealthful food and beverage purchases, poor diets, and high risk of obesity. However, information on how foods and beverages are marketed at small stores is limited. The objective of this study was to examine advertisements and product placements for healthful and less healthful foods and beverages at small stores in Minneapolis–St. Paul, Minnesota.

**Methods:**

We conducted in-store audits of 119 small and nontraditional food retailers (corner/small grocery stores, food–gas marts, pharmacies, and dollar stores) randomly selected from licensing lists in Minneapolis–St. Paul in 2014. We analyzed data on exterior and interior advertisements of foods and beverages and product placement.

**Results:**

Exterior and interior advertisements for healthful foods and beverages were found in less than half of stores (exterior, 37% [44 of 119]; interior, 20% [24 of 119]). Exterior and interior advertisements for less healthful items were found in approximately half of stores (exterior, 46% [55 of 119]); interior, 66% [78 of 119]). Of the 4 store types, food–gas marts were most likely to have exterior and interior advertisements for both healthful and less healthful items. Corner/small grocery stores and dollar stores had fewer advertisements of any type. Most stores (77%) had at least 1 healthful item featured as an impulse buy (ie, an item easily reached at checkout), whereas 98% featured at least 1 less healthful item as an impulse buy.

**Conclusion:**

Findings suggest imbalanced advertising and product placement of healthful and less healthful foods and beverages at small food stores in Minneapolis–St. Paul; less healthful items were more apt to be featured as impulse buys. Future interventions and polices should encourage reductions in advertisements and impulse-buy placements of unhealthful products, particularly in food–gas marts, and encourage advertisements of healthful products.

## Introduction

Local food environments can contribute to an unhealthful diet and obesity through increased access to energy-dense, nutrient-poor foods (1–5). The food environment not only comprises food retailers and food products but the marketing that may influence people to buy certain foods (6,7). Featured advertising, product displays, in-store promotions, and point-of-purchase product placement (ie, impulse buys) influence what people buy from food retailers (1,6–8).

Attention has focused recently on small urban food stores, especially convenience stores, for opportunities to promote health and healthful food availability (9,10). Although supermarkets still have a large portion (64%) of the market share of food consumed at home (6,11), small food stores have become frequent food sources, especially for urban residents (2,12,13). In addition, nontraditional small food stores (eg, food–gas marts, dollar stores, pharmacies), an important source of exposure to food for urban residents, have received attention (14,15).

Frequent shopping at small food stores is associated with unhealthful food purchases, low vegetable intake, and high risk of obesity (12,16–19), possibly in part because the supply of healthful foods in small food stores is limited (9,14,20,21). For example, in a study of small food stores in 4 urban US areas, only 50% of stores sold at least 1 kind of fresh fruit or vegetable (9,21). Another study found that the healthfulness of foods offered by small food stores was significantly lower than the healthfulness of foods offered by medium-sized grocers and supermarkets (2). In a study conducted in Minneapolis–St. Paul in 2014, only one-third of a sample of small food stores stocked 1 or more kinds of fresh vegetables and only one-quarter of stores stocked whole-grain–rich products (20).

In addition to the lack of availability of healthful food and beverage options in small food stores, food marketing may also contribute to customers’ purchasing decisions. However, few studies have examined the marketing, including the advertisements, product placement, and pricing strategies, of healthful and less healthful foods in these small food retailers (6,7,9). In a study published in 2011, most (97%) small food stores had some form of food and beverage advertising, either outdoors or near checkout counters. However, 94% of the advertisements were for less healthful products (eg, soda, beer, chips, and prepared foods), and only 36% were for healthful products (eg, milk, juice, and produce) (9). In the same study, nearly all stores displayed less healthful foods near the cash register as impulse buys, with only 46% of stores placing healthful products as impulse buys. These placement strategies may have the most influence on customers’ purchasing decisions (8,22). Placing products on checkout aisles and near registers can increase purchases of certain products, such as candy, sugar-sweetened beverages, and energy drinks (6,8,23). In a national sample of convenience and other retail food stores, 88% of stores displayed candy near the checkout, 29% displayed sugar-sweetened beverages, and 14% displayed fresh fruits and vegetables (7). In addition to showing that the range of retailers visited by consumers for food purchases is growing (eg, food–gas marts, dollar stores, pharmacies), the findings support the need to assess the effects of food advertisements and product placement in small food stores. Understanding the marketing environment in small food stores will assist researchers, decision makers, and other stakeholders in developing nutrition-based interventions, especially at corner stores, to improve food access and diet patterns in communities. Several corner store interventions that focus on in-store promotion or placement of healthful foods, along with other intervention strategies, have been implemented in the United States and suggest that marketing and displaying healthful foods and beverages may increase sales of those items (24,25). However, the lack of data on other types of small food retailers (eg, dollar stores, pharmacies, and food–gas marts) persists.

The aim of our study was to analyze data on exterior and interior advertisements of foods and beverages and product placement in a sample of food stores in Minneapolis–St. Paul, Minnesota. This study had 2 objectives: 1) to characterize the presence of advertisements for healthful and less healthful foods and beverages and 2) to describe the product placement of items for impulse buys. We also assessed differences in marketing strategies among corner/small grocery stores, food–gas marts, dollar stores, and pharmacies. 

## Methods

This study involved secondary data analyses of formative, pre-baseline data on food retailers collected for the Staple Food Ordinance Evaluation (STORE) study, which examined the impact of policy change on access to healthful food, particularly food sold in small and nontraditional food stores (14,20). The institutional review board of the University of Minnesota approved all study protocols.

### Study sample

Food retailers (N = 841) were randomly selected for study inclusion in the STORE study from lists of licensed grocery retailers obtained from the Minneapolis Health Department, which regulates Minneapolis retail licensing, and the Minnesota Department of Agriculture, which regulates St. Paul licensing. Stores were excluded from the sample if they 1) were supermarkets (n = 16), 2) were authorized for the Women, Infants, and Children (WIC) program (n = 175), 3) had street address problems that had been identified before the study began (n = 15), or 4) were not expected to stock an array of staple foods (eg, stores in the core downtown commercial district, liquor stores, specialty shops, other limited-ranged vendors [eg, meat or produce only], and stores with 100 square feet or less of retail space) (n = 325). Of the 310 eligible stores, a data collection team visited a random selection of 172 stores and completed store audits in 119 stores (60 in Minneapolis, 59 in St. Paul). Of the 53 stores where audits were not completed, 23 were deemed as ineligible when a researcher visited the store in-person (eg, because of new participation in WIC), 6 were out of business, one was under renovation, 10 could not be located at the listed address, and 13 refused to participate. The final analytic sample was 119 stores with completed store audits. Further details of the sample are published elsewhere (14,20).

### Store audits

Store audits were conducted on weekdays between 9:45 AM and 4:30 PM. In teams of 2, data collectors entered stores, identified themselves, and asked for permission to conduct the audit. All stores invited to participate received a mailed letter in advance describing the study.

#### Exterior and interior advertisements

The presence of advertisements for healthful and less healthful items was recorded as part of a larger, more extensive store audit to assess factors such as food availability and pricing. Data were collected according to the CX^3^, a previously developed food availability and marketing survey (26), which demonstrated good interrater and test–retest reliability. Our study team modified the survey to focus only on recording the presence of at least 1 advertisement of any size or product on the exterior or interior of a store.

For assessing exterior advertisements, data collectors recorded whether images of healthful foods or less healthful foods were present (yes/no) on storefront doors or windows. Images were defined to include brands, logos, or texts of specified food items (specified by the STORE study) and well-known products (eg, Coca Cola, Pepsi). For interior advertisements, data collectors recorded whether images of healthful or less healthful foods were present next to checkout, below checkout, on the floor next to checkout, or hanging from the ceiling. Food and beverage advertisements are found in these areas (9). A composite variable was created to measure the presence of advertisements in any of the 4 interior locations. Healthful foods were defined (in the STORE study) as fruits and vegetables, whole grains, beans, nuts and seeds, non-fat or low-fat milk products, lean meat, poultry, fish, and black coffee. These items had minimal or no added fats, sugars, or sweeteners. We defined less healthful foods as high-calorie, low-nutrient items, including alcoholic beverages (exterior advertisements only); soft drinks and other sweetened beverages, including diet drinks; sweet desserts and highly sugared cereals; chips and other salty snacks; most solid fats; fried foods; and other foods with high amounts of sugar, fat, or sodium.

#### Product placement

To assess product placement, defined as the placement of items to promote impulse buys, data collectors recorded the following: 1) the presence of less healthful items, such as gumball machines, candy, soda, chips, and others (eg, energy drinks, beef jerky, cookies, donuts, ice cream, pastries) within reach of the cash register, and 2) the presence of healthful food items within reach of the cash register. Healthful food items included trail mix or granola (no added sugar), bagged nuts or seeds (no added sugar), fresh fruit, bottled water, and others, such as 100% fruit juice. We created composite variables to assess the presence of product placement for both healthful and less healthful items.

Interrater reliability was assessed in 33 stores. Agreement was excellent for the presence of at least 1 exterior advertisement for a healthful or less healthful item (100%), at least 1 interior advertisement for a healthful or less healthful item (91%), and at least 1 product placement for a less healthful item (100%). Agreement was good for the presence of at least 1 product placement for a healthful item (88%). Agreement for individual items ranged from 70% to 100%.

### Analysis

Descriptive statistics on store characteristics (store type, number of cash registers, and number of aisles), advertisements, and product placement were calculated to describe data on all stores combined and stores stratified by type (corner/small grocery store, food–gas mart, dollar store, or pharmacy). The data on number of cash registers and store aisles were highly skewed, so we used the nonparametric Mann–Whitney test to test differences across store types. Differences in categorical variables (presence of advertisements for healthful or less healthful items and product placement) by store type were tested using a 3-degrees-of-freedom χ^2^ test and single-degree-of-freedom pairwise comparisons. Results with a *P* value < .05 were considered significant. In a summary assessment, we classified the data for all 119 stores according to type of advertising or promotion (exterior, interior, or product placement) and whether or not the type was for healthful items, less healthful items, both healthful and less healthful items, or none. Analyses were conducted in Stata version 13.1 (StataCorp LP).

## Results

Of the 119 stores studied, 46 (39%) were corner/small grocery stores, 51 (43%) were food–gas marts, 9 (8%) were dollar stores, and 13 (11%) were pharmacies (Table 1). The median number of cash registers and aisles varied by store type, with dollar stores (13 aisles) and pharmacies (9 aisles) having significantly more aisles than corner/small grocery stores (4 aisles) and food–gas marts (5 aisles). Pharmacies had significantly more cash registers (7 registers) than other store types. Most stores accepted benefits from the Supplemental Nutrition Assistance Program (SNAP), regardless of store type. Nearly one-quarter of food–gas marts (26%; 13 of 50) and pharmacies (23%; 3 of 13) were open for 24 hours per day, whereas no corner/small grocery stores or dollar stores were open for 24 hours.

**Table 1:** Characteristics of Small Stores That Sell Food in Minneapolis–St. Paul, 2014

Characteristics	All Stores	Corner/Small Grocery Store	Food–Gas Mart	Dollar Store	Pharmacy	*P* Value^a^
No. (%)	119 (100)	46 (39)	51 (43)	9 (8)	13 (11)	—
No. of cash registers, median (range)	2 (1–11)	1 (1–11)^b^	2 (1–3)^c^	3 (2–5)^d^	7 (1–10)^e^	<.001
No. of aisles, median (range)	5 (1–27)	4 (1–25)^b^	5 (1–11)^b^	13 (3–27)^c^	9 (2–19)^c^	<.001
Accepts electronic benefits transfer (EBT), numerator/denominator (%)	91/117 (78)	31/45 (69)^b^	39/50 (78)^b^^,^^c^	9/9 (100)^d^	12/13 (92)^c^^,^^d^	.10
Sells tobacco, numerator/denominator (%)	88/119 (74)	23/46 (50)^b^	51/51 (100)^c^	3/9 (33)^b^	11/13 (85)^c^	<.001
Open 24 h, numerator/denominator (%)	16/117 (14)	0/45 (0)^b^	13/50 (26)^c^	0/9 (0)^b^	3/13 (23)^b^^,^^c^	.001

a From equality of medians or (for proportions) χ^2^ test.

b,c,d,e Pairwise comparisons that were significantly different from one another are indicated as follows: when 2 values within a row do not share a common superscript, they are significantly different, whereas when 2 values within a row do share a common superscript, they are not significantly different. Significance (*P* < .05) determined by Mann–Whitney test for median and Wald test for proportions.

A little more than half of all stores (57%; 68 of 119) had at least 1 exterior advertisement of foods or beverages: 37% (44 of 119) had advertisements of healthful items, and 46% (55 of 119) had advertisements of less healthful items (Table 2). A significantly higher percentage of food–gas marts had at least 1 healthful (55%; 28 of 51) and at least 1 less healthful exterior advertisement (75%; 38 of 51) compared with corner/small grocery stores (healthful, 17% [8 of 46]; less healthful, 35% [16 of 46]). In addition, a significant higher percentage of pharmacies had at least 1 healthful exterior advertisement (46%; 6 of 13) compared with corner stores (17%; 8 of 46). Dollar stores (22%; 2 of 9) were least likely to have at least 1 exterior advertisement. Similarly, a significantly higher percentage of food–gas marts had at least 1 interior advertisement for healthful (39%; 20 of 51) or less healthful (88%; 45 of 51) items compared with corner/small grocery stores (healthful, 2% [1 of 46]; less healthful, 43% [20 of 46]).

**Table 2:** Advertisements and Product Placements for Healthful^a^ and Less Healthful^b^ Food and Beverages in Small Stores in Minneapolis–St. Paul, 2014

Type	No. (%)					*P* Value^c^
	All Stores (N = 119)	Corner/Small Grocery Store (n = 46)	Food–Gas Mart (n = 51)	Dollar Store (n = 9)	Pharmacy (n = 13)	
**Advertisements**						
**Exterior advertisements**						
At least 1	68 (57)	18 (39)	41 (80)	2 (22)	7 (54)	<.001
Advertisements for healthful foods and beverages	44 (37)	8 (17)^d^	28 (55)^e^	2 (22)^d^^,^^f^	6 (46)^e^^,^^f^	.001
Advertisements for less healthful foods and beverages	55 (46)	16 (35)^d^	38 (75)^e^	0 (0)^f^	1 (8)^f^	<.001
**Interior advertisements**						
**At least 1 interior advertisement**	82 (69)	21 (46)	47 (92)	7 (78)	7 (54)	<.001
**Healthful^a^ interior advertisement**						
At least 1	24 (20)	1 (2)^d^	20 (39)^e^	0 (0)^d^	3 (23)^d^^,^^e^	<.001
Next to the cash register	7 (6)	0 0)^d^	6 (12)^e^	0 (0)^d^	1 (8)^d^^,^^e^	.08
Below the cash register	2 (2)	0 (0)^d^	1 (2)^d^	0 (0)^d^	1 (8)^d^	.28
On the floor	6 (5)	0 (0)^d^	5 (10)^e^	0 (0)^d^	1 (8)^d^^,^^e^	.14
Hanging from the ceiling	16 (13)	1 (2)^d^	15 (29)^e^	0 (0)^d^	0 (0)^d^	<.001
**Less healthful^b^ interior advertisement**						
At least 1	78 (66)	20 (43)^d^	45 (88)^e^	7 (78)^e^^,^^f^	6 (46)^d^^,^^f^	<.001
Next to the cash register	51 (43)	11 (24)^d^	31 (61)^e^	5 (56)^d^^,^^e^	4 (31)^d^	.002
Below the cash register	17 (14)	3 (7)^d^	13 (25)^e^	0 (0)^d^	1 (8)^d^^,^^e^	.02
On the floor	36 (30)	7 (15)^d^	23 (45)^e^	2 (22)^d^^,^^e^	4 (31)^d^^,^^e^	.02
Hanging from the ceiling	15 (13)	2 (4)^d^	12 (24)^e^	0 (0)^d^	1 (8)^d^^,^^e^	.02
**Product Placement^g^**						
**At least 1^g^**	117 (98)	44 (96)	51 (100)	9 (100)	13 (100)	.36
**Healthful^a^ product placement^g^**						
At least 1	92 (77)	36 (78)^d^	40 (78)^d^	5 (56)^d^	11 (85)^d^	.41
Trail mix or granola (no added sugar)	18 (15)	5 (11)^d^	9 (18)^d^	0 (0)^e^	4 (31)^d^	.17
Nuts or seeds (no added sugar)	46 (39)	15 (33)^d^	24 (47)^d^	2 (22)^d^	5 (38)^d^	.35
Fruit	29 (24)	9 (20)^d^	18 (35)^d^	2 (22)^d^^,^^e^	0 (0)^e^	.04
Water	26 (22)	11 (24)^d^	4 (8)^e^	5 (56)^d^	6 (46)^d^	.001
Other healthful food items	22 (18)	8 (17)^d^	9 (18)^d^	0 (0)^e^	5 (38)^d^	.14
**Less healthful^b^ product placement^g^**						
At least 1	117 (98)	44 (96)^d^	51 (100)^d^	9 (100)^d^	13 (100)^d^	0.36
Gumball machine	15 (13)	6 (13)^d^	4 (8)^d^	5 (56)^e^	0 (0)^f^	<.001
Candy	112 (94)	40 (87)^d^	50 (98)^e^	9 (100)^e^	13 (100)^e^	.07
Soda	55 (46)	16 (35)^d^	21 (41)^d^	8 (89)^e^	10 (77)^e^	.002
Chips	75 (64)	25 (56)^d^	39 (76)^e^	5 (56)^d^^,^^e^	6 (46)^d^^,^^e^	.08
Other less healthful food items	109 (92)	39 (85)^d^	49 (96)^d^^,^^e^	8 (89)^d^^,^^e^	13 (100)^e^	.15

a Healthful foods and beverages were defined as fruits and vegetables, whole grains, beans, nuts and seeds, non-fat/low-fat milk products, lean meat, poultry, fish, and unsweetened black coffee, according to definitions in the Staple Food Ordinance Evaluation (STORE) study (15,21).

b Less healthful food were high-calorie, low-nutrient foods and beverages, including alcoholic beverages (exterior advertisements only); soft drinks and other sweetened beverages including diet drinks, sweet desserts and highly sugared cereals, chips and other salty snacks, most solid fats, fried foods, and other foods with high amounts of sugar, fat and/or sodium.

c Significant difference among groups determined by χ^2^ test.

d,e,f Pairwise comparisons that were significantly different from one another are indicated as follows: when 2 values within a row do not share a common superscript, they are significantly different, whereas when 2 values within a row do share a common superscript, they are not significantly different. Significance (*P* < .05) determined by *t* test.

g Product placement defined as the placement of items to promote impulse buys: food items placed near the cash register, including spaces on and below the counter and displays surrounding the areas where customers wait in line.

Overall, 77% (92 of 119) of stores had at least 1 healthful product placement, whereas nearly all stores (98%; 117 of 119) had at least 1 less healthful product placement (Table 2). Of these latter 117 stores, 46% promoted beef jerky, 35% promoted energy drinks, and 27% promoted cookies as impulse buys. We observed differences in type of product placements by type of store. For example, a significantly lower percentage of food–gas marts (8%; 4 of 51) promoted water as an impulse buy than corner/small grocery stores (24%; 11 of 46), dollar stores (56%; 5 of 9), or pharmacies (46%; 6 of 13). In addition, a significantly higher percentage of food–gas marts promoted candy (98%; 50 of 51) and chips (76%; 39 of 51) as impulse buys than corner/small grocery stores (candy, 87% [40 of 46]; chips, 56% [25 of 46]). Lastly, a higher percentage of dollar stores (89%; 8 of 9) and pharmacies (77%; 10 of 13) than corner/small grocery stores (35%; 16 of 46) promoted soda as an impulse buy.

In stratified analyses, we did not find any significant differences in advertisements or product placement by whether the store accepted SNAP. However, we did observe significant positive associations between advertisements and product placements by whether the store sold tobacco.

In our summary assessment, 43% (51 of 119) of stores did not have any exterior advertisements, and 31% (37 of 119) did not have any interior advertisements (Table 3). Of stores that had advertisements, few displayed advertisements for healthful items. Only 2% of stores had no product placements; of stores that had them, no product placements promoted only healthful items.

**Table 3:** Summary Profile of Small Stores (n = 119) Participating in Study of Advertising and Product Placement of Healthful and Less Healthful Food and Beverage Items in Minneapolis–St. Paul, 2014

Type of Advertising or Product Placement^a^	Exterior Advertisements, No. (%)	Interior Advertisements, No. (%)	Product Placement^a^, No. (%)
None	51 (43)	37 (31)	2 (2)
Only healthful	13 (11)	4 (3)	0 (0)
Only less healthful	24 (20)	58 (49)	25 (21)
Both healthful and less healthful	31 (26)	20 (17)	92 (77)
Total	119 (100)	119 (100)	119 (100)

a Product placement defined as the placement of items to promote impulse buys: food items placed near the cash register, including spaces on and below the counter and displays surrounding the areas where customers wait in line.

## Discussion

Overall, we found that a low percentage (37%) of all stores had exterior advertisements for healthful foods or beverages. In contrast, 46% of stores had at least 1 type of advertisement for less healthful items. For exterior advertisements of both healthful and less healthful items, food–gas marts were significantly more likely to have at least 1 food or beverage advertisement, compared with corner/small grocery stores or dollar stores. Two-thirds of stores included interior advertisements for less healthful items; nearly all stores (98%) placed some type of less healthful item so as to encourage an impulse buy.

Possible explanations for our findings are rooted in the very purpose of food marketing. Retail stores, including supermarkets, supercenters, and small stores are in the business of prompting people to purchase foods (6) to make a profit. Typical ways to promote food and beverage products are featured advertisements, product displays, and point-of-purchase availability (1). The food industry pays substantially to place products in stores, especially near the checkout (6). However, the impact of these promotional strategies varies by consumer and store type (1,27). For instance, customers may associate certain store types with certain types of food items, regardless of the marketing features present; for example, at food–gas marts people may expect advertisements for less healthful items and go there to purchase them. Customers’ perceptions of store features are influenced by their goals (8). Industry has traditionally focused heavily on promoting and selling less healthful foods and beverages; more focus is needed on how to promote healthful products that can be profitable for retailers and beneficial to the health of customers.

To remedy the imbalance of healthful and less healthful food advertisements and product placement in stores, the public health community has recommendations. The Center for Science in the Public Interest recommends that food stores adopt nutrition standards for food sold at checkouts, including selling at checkout only nonfood items or healthful food and beverage options (6). Given evidence that placing products in checkout areas increases purchases and that checkout areas are dominated by unhealthful options (7), leading health organizations, particularly those working with food retailers, may consider developing marketing strategies that set nutrition standards for retail checkouts. These and other product placement strategies have been implemented in some stores across the United States (8,23–25) and are recommended for broader adoption (22).

Unfortunately, such marketing strategies may not work for all small retailers, especially if the strategies lead to a decrease in profits. Because small food store owners and managers may have limited control over how contracts with food manufacturers are set up and the conditions of these contracts (eg, requirements for product placement), there needs to be an effort to empower and equip these owners and managers to negotiate such contracts with food manufacturers, particularly in ways that could incentivize stores to promote healthful food products.

Our study has several limitations. First, the data collected were for only 1 urban geographic region, and thus the data may have limited generalizability to other communities. Although our sample size was comparable with the samples sizes in previous corner store studies, the sociodemographic makeup, small store characteristics, and government regulations and policies in Minneapolis–St. Paul may be different from those in other US cities and may have influenced food marketing in our study area in ways that would not apply in other cities. Second, only data on the existence of at least 1 advertisement were collected to describe advertisements for healthful and less healthful items. We did not capture data on the overall composition and type of foods and beverages advertised inside or outside stores, nor did we capture data on the number of items representing all advertisements for healthful and less healthful items. Thus, the magnitude of differences between healthful and less healthful advertisements could be masked by our dichotomized variables. Third, our study relied on previous classifications of healthful and less healthful items; distinguishing between healthful and less healthful can be difficult (eg, nuts were considered healthful, even when they were high in sodium). In future studies, the classification of healthful and less healthful foods should be improved. Fourth, our analyses did not account for neighborhood characteristics. Less healthful items may be advertised more in low-socioeconomic neighborhoods than neighborhoods with greater prosperity (7). Future work should address differences by neighborhood characteristics, including poverty level and race/ethnicity.

Despite these limitations, to our knowledge this is one of a few studies that describes interior and exterior food advertising and marketing in small food stores. It expands the universe of small stores sampled to include corner/small grocery stores, food–gas marts, pharmacies, and dollar stores. Previous work on advertising and promotions examined only selected convenience stores surrounding urban schools (9).

Our findings suggest imbalanced advertising and product placement of healthful and less healthful foods and beverages at small food stores, with less healthful foods more apt to be advertised and placed by the checkout for impulse buying. Future interventions and polices should encourage reductions in advertisements and impulse-buy placements of less healthful products, particularly in food–gas marts, and encourage advertisements of healthful products. The adoption of healthful checkout aisles by large food retailers is both feasible and well-received by customers; future work needs to explore whether this strategy can be effectively implemented in small food stores (6). ChangeLab Solutions drafted model language for use by communities interested in adopting ordinances for healthful checkout aisles; this language urges policy action to require all retail food stores to provide healthful checkout aisles (28). Ultimately, all stakeholders — citizens, food retailers, manufacturers, policymakers, and the public health community — should work together on solutions to promote healthful food purchases at small urban food stores without reducing the financial viability of stores.
